# Inhibition of PFKFB3 suppresses osteoclastogenesis and prevents ovariectomy‐induced bone loss

**DOI:** 10.1111/jcmm.14912

**Published:** 2019-12-27

**Authors:** Jia Wang, Hanfeng Guan, Hui Liu, Zuowei Lei, Honglei Kang, Qian Guo, Yimin Dong, Huiyong Liu, Yunlong Sun, Zhong Fang, Feng Li

**Affiliations:** ^1^ Department of Orthopedics Tongji Hospital Tongji Medical College Huazhong University of Science and Technology Wuhan China

**Keywords:** glycolysis, MAPKs, NF‐κB, osteoclast, PFKFB3

## Abstract

Osteoclasts are multinucleated cells derived from the monocyte/macrophage cell lineage under the regulation of receptor activator of nuclear factor‐κB ligand (RANKL). In previous studies, stimulation by RANKL during osteoclastogenesis was shown to induce a metabolic switch to enhanced glycolytic metabolism. Thus, we hypothesized that blockage of glycolysis might serve as a novel strategy to treat osteoclast‐related diseases. In the present study, 6‐phosphofructo‐2‐kinase/fructose‐2,6‐bisphosphatase 3 (PFKFB3), an essential regulator of glycolysis, was up‐regulated during osteoclast differentiation. Genetic and pharmacological inhibition of PFKFB3 in bone marrow‐derived macrophages suppressed the differentiation and function of osteoclasts. Moreover, intraperitoneal administration of the PFKFB3 inhibitor PFK15 prevented ovariectomy‐induced bone loss. In addition, glycolytic activity characterized by lactate accumulation and glucose consumption in growth medium was reduced by PFKFB3 inhibition. Further investigation indicated that the administration of L‐lactate partially reversed the repression of osteoclastogenesis caused by PFKFB3 inhibition and abrogated the inhibitory effect of PFK15 on the activation of NF‐κB and MAPK pathways. In conclusion, the results of this study suggest that blockage of glycolysis by targeting PFKFB3 represents a potential therapeutic strategy for osteoclast‐related disorders.

## 1 INTRODUCTION

Bone is a dynamic organ that has a delicate balance between osteoblast‐mediated bone formation and osteoclast‐mediated bone resorption.^1^ Excessive bone resorption can lead to imbalance of bone turnover and result in various bone diseases, such as osteoporosis. Osteoporosis is a metabolic skeletal disease characterized by decreased mineral density and increased susceptibility to fracture.^2^ Approximately 200 million people around the world suffer from this disease, causing a heavy burden on public health.^3^ Although several anti‐osteoporotic drugs are available, the limitations and side effects of them cannot be ignored.^4^ Exploring novel targets or mechanisms underlying osteoclastic bone resorption may improve therapeutic benefits and reduce side effects in the treatment of osteoporosis, and other disorders caused by osteoclasts.

Osteoclasts are multinucleated cells derived from the monocyte/macrophage cell lineage under the regulation of two crucial cytokines, macrophage colony stimulating factor (M‐CSF) and receptor activator of nuclear factor‐κB (RANK) ligand (RANKL).^5^ M‐CSF is responsible for vitality and proliferation of osteoclast precursors, whereas RANKL induces the differentiation of osteoclasts. RANKL exerts its effect by binding to its receptor RANK to recruit the tumour necrosis factor receptor associated factor 6 (TRAF6). Consequently, mitogen‐activated protein kinase (MAPK) and nuclear factor‐κB (NF‐κB) pathways are activated, eventually leading to the induction of transcription factors such as c‐FOS and nuclear factor of activated T cells c1 (NFATc1).^6^, ^7^

Osteoclastogenesis is an active metabolic process, and previous studies have shed light on the metabolic changes during RANKL‐induced osteoclastogenesis. For example, the metabolic enzymes involved in glycolysis and mitochondrial oxidative phosphorylation were reportedly up‐regulated during osteoclast differentiation.^8^, ^9^ Additionally, stimulation by RANKL during osteoclastogenesis was shown in previous studies to induce a metabolic switch to both enhanced glycolysis and accelerated mitochondrial respiration.^10^, ^11^ Taken together, these results indicate that both glycolysis and mitochondrial respiration might participate in the regulation of osteoclastogenesis and can serve as potential targets for osteoclast‐related diseases. The importance of mitochondrial respiration during RANKL‐induced osteoclastogenesis has been well‐established. For example, an abundance of mitochondria has been identified as the typical morphological feature of osteoclasts,^12^ and inhibitors of mitochondrial complexes such as rotenone and antimycin A were shown in previous studies to significantly inhibit osteoclast differentiation.^10^, ^13^ However, a little is known about the role of glycolysis during RANKL‐induced osteoclastogenesis.

Glycolysis is the metabolic pathway that converts glucose into pyruvate eventually resulting in the production of L‐lactate. Glycolytic metabolism supplies energy for multiple physiological phenomena and regulates physiological processes by the formation of specific glycolytic intermediates. The conversion of fructose‐6‐phosphate (F6P) to fructose‐1,6‐bisphosphate (F1,6BP) by 6‐phosphofructo‐1‐kinase (PFK‐1) is a rate‐limiting and irreversible step of glycolysis, suggesting that PFK‐1 serves as an essential regulator in glycolytic metabolism.^14^ Synthesis and degradation of fructose‐2,6‐bisphosphate (F2,6BP), the most potent allosteric activator of PFK‐1, are under the control of 6‐phosphofructo‐2‐kinase/fructose‐2,6‐bisphosphatase (PFKFB).^15^ PFKFB is a bifunctional enzyme with both kinase and phosphatase activities. Four PFKFB isoenzymes have been identified (PFKFB1‐4), among which PFKFB3 has the highest kinase/phosphatase activity ratio.^16^ Thus, PFKFB3 can promote glycolytic activity by producing F2,6BP with its high kinase activity. Previous studies concerning PFKFB3 mainly focused on its role in cancer. Li et al^17^ showed that pharmacological inhibition of PFKFB3 with its inhibitor PFK15 suppressed the growth of head and neck squamous cell carcinoma by reducing glycolytic flux. In addition, PFKFB3 has been reported as a mediator of circadian control of cancer growth.^18^ Moreover, based on the results of a recent study, PFKFB3 radiosensitized cancer cells and suppressed homologous recombination.^19^ However, the function of PFKFB3 during osteoclastogenesis remains obscure. In the present study, genetic and pharmacological inhibition of PFKFB3 was utilized to investigate the function of PFKFB3 in osteoclast differentiation. Furthermore, an ovariectomized murine model was used to further elucidate the contribution of PFKFB3 to the pathogenesis of osteoclast‐related diseases.

## 2 MATERIALS AND METHODS

### 2.1 Reagents and antibodies

PFK15 was purchased from Selleck Chemicals. Recombinant murine M‐CSF and recombinant murine RANKL were obtained from PeproTech. Antibodies against P65 (#8242), p‐P65 (#3033), IκBα (#4812), p‐IκBα (#2859), P38 (#8690), p‐P38 (#4511), JNK (#9252), p‐JNK (#4668), ERK (#4695), p‐ERK (#4370), NFATc1 (#8032) and c‐FOS (#2250) were purchased from Cell Signaling Technology. Antibodies against PFKFB3 (#13763‐1‐AP), MMP9 (#10375‐2‐AP) and β‐Actin (#20536‐1‐AP) were obtained from Proteintech Group. L‐lactate and other reagents were purchased from Sigma‐Aldrich.

### 2.2 Bone marrow‐derived macrophage isolation and osteoclast differentiation

Bone marrow‐derived macrophages (BMMs) were isolated from the long bones of eight‐week‐old male C57BL/6 mice. Briefly, the tibias and femurs were separated from the mice and the bone marrow cells were flushed out from marrow cavities. The cells were cultured in α‐MEM medium containing 10% foetal bovine serum (FBS) and M‐CSF (30 ng/mL) for 16 hours. Next, the medium containing floating cells was transferred to a new culture dish. After 2 days, the medium was discarded and adherent cells were considered as BMMs for further use.

For osteoclast differentiation, BMMs were seeded in 96‐well plates at a density of 1.5 × 10^4^ cells/well and cultured in osteoclastogenic medium (α‐MEM medium containing 10% FBS, 30 ng/mL M‐CSF and 75 ng/mL RANKL) for 5 days to differentiate into osteoclasts. Next, TRAP staining was performed. TRAP‐positive multinucleated cells with three or more nuclei were identified as mature osteoclasts.

### 2.3 Adenoviral transduction

Adenoviruses encoding murine PFKFB3 and control adenoviruses were obtained from Vigene Biosciences. Four short hairpin RNAs (shRNAs) were designed and packaged in one vector targeting different regions of PFKFB3. The following sequences were used: shRNA1, 5′‐GGATAGGTGTTCCAACGAAAGTTCAAGAGACTTTCGTTGGAACACCTATCCTTTTTT‐3′, shRNA2, 5′‐GCGAGAATGAGTACAACTTGCTTCAAGAGAGCAAGTTGTACTCATTCTCGCTTTTTT‐3′, shRNA3, 5′‐GGAGCCTGTGATCATGGAATTTTCAAGAGAAATTCCATGATCACAGGCTCCTTTTTT ‐3′ and shRNA4, 5′‐GCCTCGCATCAACAGCTTTGATTCAAGAGATCAAAGCTGTTGATGCGAGGCTTTTTT ‐3′.

For adenoviral transduction, BMMs were isolated as previously described and incubated in α‐MEM medium with M‐CSF supplementation. After 24 hours, cells were washed and then cultured with adenoviral particles (50 particles per cell) for 12 hours. Knockdown effects were confirmed by qPCR and Western blotting.

### 2.4 Cell proliferation assay

Bone marrow‐derived macrophages were seeded in 96‐well plates at a density of 5 × 10^3^ cells/well and cultured in α‐MEM medium with M‐CSF supplementation for 24 hours. Then, the cells were treated with various concentrations of PFK15 for 1, 3 and 5 days. Cell viability was measured by adding cell counting kit‐8 (Boster Biotechnology) buffer to the medium and incubating at 37°C for 1 hour. Absorbance at 450 nm was detected using a microplate reader (Bio‐Tek).

### 2.5 Pit formation assay

Bone marrow‐derived macrophages were seeded into a 0.2% collagen‐gel–coated 6‐well plate and cultured in osteoclastogenic medium for 4 days. Then, the cells were dissociated from the plate using collagenase (0.2%) and equal numbers of cells were seeded onto an osteo assay surface coated with hydroxyapatite in a 96‐well plate (Corning). The cells were treated with different concentrations of PFK15 for 2 days. Finally, the cells were removed from the surface by incubating with 10% bleaching solution for 5 minutes and resorption pits were captured using a light microscope.

### 2.6 Immunofluorescence staining

Bone marrow‐derived macrophages were washed with phosphate‐buffered saline (PBS) and fixed with 4% paraformaldehyde for 15 minutes. Then, the cells were permeabilized with 0.1% Triton‐X and incubated with rhodamine‐conjugated phalloidin (Sigma‐Aldrich) for 1 hour at 25°C to stain for F‐actin. Next, the nuclei were stained with DAPI for 5 minutes after washing with PBS for three times. Fluorescence images were captured using a fluorescence microscope and analysed with ImageJ software.

### 2.7 RNA isolation and qPCR

Total RNA was isolated using TRIzol reagent (Invitrogen) according to the manufacturer's specifications. One microgram of total RNA was used to synthesize cDNA with an oligo‐dT primer and reverse transcriptase (Thermo Scientific). qPCR was performed using the mixture of cDNA, SYBR reagent (Kapa Biosystems) and specific primers shown as follows: PFKFB3, forward 5′‐CCCAGAGCCGGGTACAGAA‐3′ and reverse 5′‐GGGGAGTTGGTCAGCTTCG‐3′; NFATc1, forward 5′‐CAACGCCCTGACCACCGATAG‐3′ and reverse 5′‐GGGAAGTCAGAAGTGGGTGGA‐3′; c‐FOS, forward 5′‐CCAGTCAAGAGCATCAGCAA‐3′ and reverse 5′‐AAGTAGTGCAGCCCGGAGTA‐3′; TRAP, forward 5′‐TACCTGTGTGGACATGACC‐3′ and reverse 5′‐CAGATCCATAGTGAAACCGC‐3′; MMP9, forward 5′‐TCCAGTACCAAGACAAAGCCTA‐3′ and reverse 5′‐TTGCACTGCACGGTTGAA‐3′; GAPDH, forward 5′‐TCATTGACCTCAACTACATG‐3′ and reverse 5′‐TCGCTCCTGGAAGATGGTGAT‐3′. The relative mRNA levels of target genes were calculated by the 2^–ΔΔCT^ method using GAPDH as an internal control.

### 2.8 Western blotting

Total protein was extracted from cultured cells by using RIPA buffer supplemented with 1% proteinase inhibitor and 1% phosphotransferase inhibitor (Boster Biotechnology). Proteins were separated by 10% SDS‐PAGE gel electrophoresis and then transferred to PVDF membranes (Millipore). The membranes were blocked with 5% BSA a for 1 hour and then incubated with primary antibodies followed by washing with Tris‐buffered saline Tween 20 (TBST) for three times. Blots were then incubated with horseradish peroxidase–conjugated secondary antibodies, and the signals were detected with ECL Substrate Kit (Thermo Scientific).

### 2.9 Murine model of ovariectomy (OVX)‐induced bone loss

Twelve‐week‐old female C57BL/6 mice were provided by the Experimental Animal Centre of Tongji Medical College. All mice were housed in cages with 12‐hours light and dark cycles and were fed with food and water ad libitum. The mice were randomly divided into four equal groups (n = 7): sham‐surgery mice treated with vehicle (SHAM + VEH), sham‐surgery mice treated with PFK15 (SHAM + PFK15), OVX mice treated with vehicle (OVX + VEH), and OVX mice treated with PFK15 (OVX + PFK15, 20 mg/kg, every other day). For OVX and sham surgeries, mice were anaesthetized with pentobarbital, and incisions were made through a dorsal approach. Ovariectomy was performed by removing the bilateral ovaries, and the sham surgery was conducted by only identifying the bilateral ovaries. Three days after the surgery, mice were injected intraperitoneally with PFK15 or vehicle for 6 weeks. Finally, the mice were killed to collect serum, tibias and femurs for further experiments. All intervention procedures of the mice were approved by the Animal Care and Use Committee of Tongji Medical College.

### 2.10 Microcomputed tomography scanning and histomorphometric analysis

After removal of soft tissues, the left femur of each mouse was fixed and scanned using microcomputed tomography (μ‐CT, Scanco Medical). Scans were taken with a source voltage of 100 kV, a current of 98 μA and a voxel size of 10 μm. The three‐dimensional bone structure images and the bone structural parameters including bone volume/tissue volume (BV/TV), trabecular numbers (Tb.N), trabecular thickness (Tb.Th) and trabecular space (Tb.Sp) were reconstructed using the built‐in software.

The right femur of each mouse was fixed in 4% paraformaldehyde solution for 1 day and then decalcified with 10% EDTA for 2 weeks. Next, the decalcified femurs were embedded in paraffin and sectioned for TRAP staining.

### 2.11 Measurement of intracellular F2,6BP

Bone marrow‐derived macrophages were collected to detect F2,6BP levels using a coupled enzyme reaction as previously reported.^20^ Finally, the F2,6BP concentration was normalized to total cellular protein based on the bicinchoninic acid (BCA) assay (Beyotime) measurements according to the manufacturer's instructions.

### 2.12 L‐lactate detection assay

L‐lactate concentration in growth media was measured using a L‐Lactate Assay kit (Abcam) according to the manufacturer's protocols. L‐lactate is oxidized by lactate dehydrogenase to generate a product which interacts with a probe to produce a colour (λ = 450 nm). Briefly, the medium was collected and then incubated with reaction mix at room temperature for 30 minutes. Absorbance at a wavelength of 450 nm was measured using a microplate reader (Bio‐Tek).

### 2.13 Glucose detection assay

Glucose concentration in growth media was detected using a Glucose Assay kit (Abcam) according to the manufacturer's instructions. Glucose can be specifically oxidized by the glucose enzyme mix to generate a product which reacts with a dye to generate colour (λ = 570 nm). In brief, the medium was collected and then incubated with reaction mix at 37°C in the dark for 30 minutes. Absorbance at a wavelength of 570 nm was assessed using a microplate reader (Bio‐Tek).

### 2.14 Oxygen consumption assay

Oxygen consumption was measured using an oxygen consumption assay kit (Abcam) following the manufacturer's instructions. The assay is based on the ability of oxygen to quench the excited state of oxygen consumption reagent presents in the kit, and the consumption of oxygen is reflected by an increase in phosphorescence signal. Briefly, the culture medium was added with oxygen consumption reagent and sealed with high‐sensitivity mineral oil to limit diffusion of ambient oxygen. The fluorescent signal (excitation of 380 nm/emission of 650 nm) was detected using a microplate reader (Bio‐Tek).

### 2.15 Statistical analysis

Data are expressed as the means ± standard deviation (SD) of at least three independent experiments. Unpaired Student's *t* tests were used to determine the significance of differences between two groups, and one‐way ANOVA was used for multiple comparisons. Statistical significance was considered as *P* < .05.

## 3 RESULTS

### 3.1 Inhibition of PFKFB3 suppresses osteoclastogenesis

**Figure 1:**
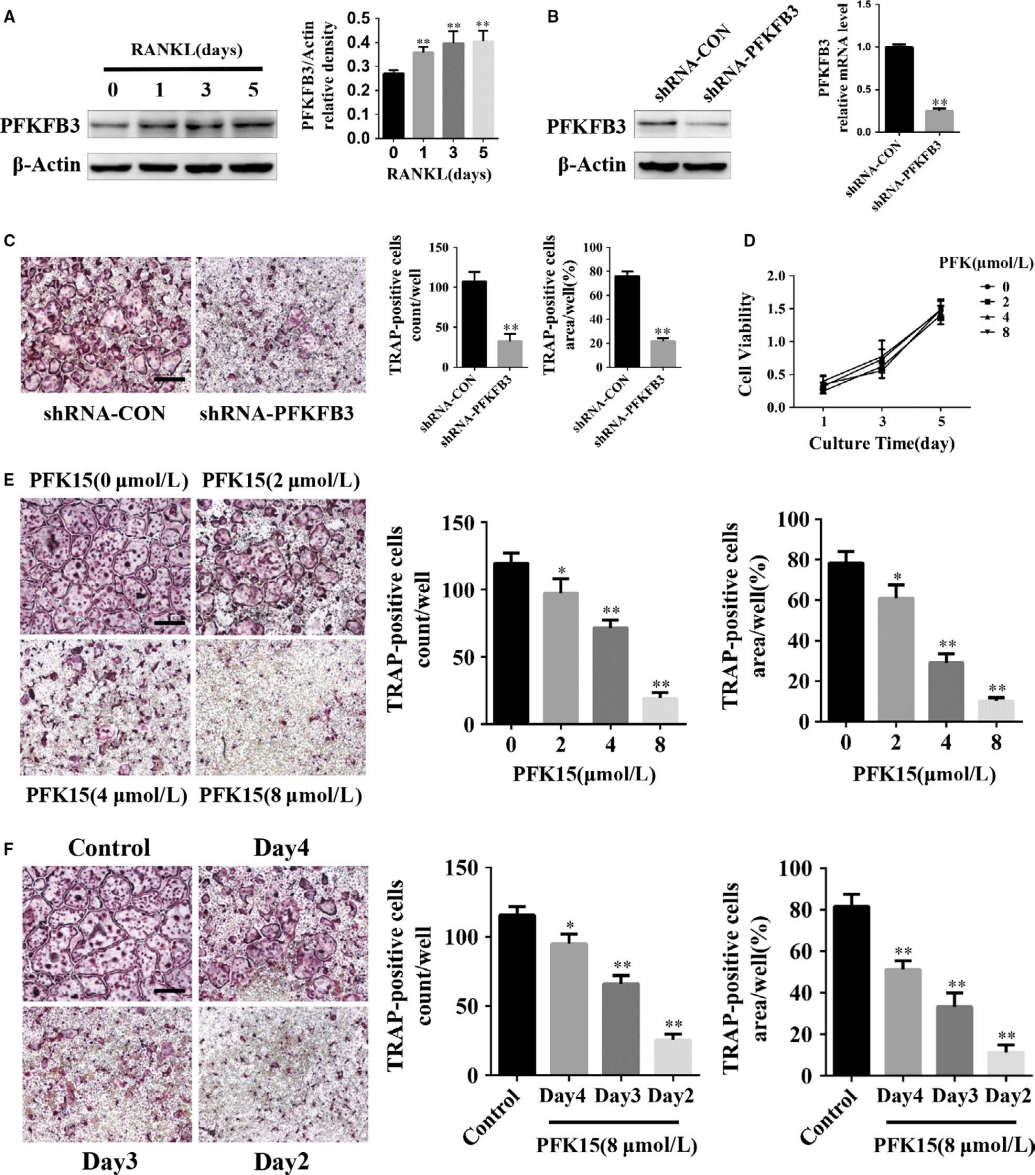
PFKFB3 is up‐regulated during osteoclast differentiation, and inhibition of PFKFB3 suppresses osteoclastogenesis. A, BMMs were treated with RANKL (75 ng/mL) for the indicated times. The protein level of PFKFB3 was analysed by Western blotting. B, Knockdown efficacy of adenoviral transduction. BMMs were infected with the adenovirus carrying PFKFB3 shRNA or the control adenovirus with M‐CSF supplementation for 3 d, and the expression of PFKFB3 was measured using qPCR and Western blotting. C, BMMs were infected with the adenovirus carrying PFKFB3 shRNA or the control adenovirus and then cultured in the presence of M‐CSF and RANKL for 5 d. TRAP staining was performed, and TRAP‐positive cells with three or more nuclei were quantified. D, BMMs were cultured with M‐CSF and various concentrations of PFK15 for different time periods. Proliferation of BMMs was detected with CCK8. E, BMMs were cultured with various concentrations of PFK15 in the presence of M‐CSF and RANKL for 5 d. TRAP‐positive multinucleated osteoclasts were counted. F, BMMs were cultured with M‐CSF and RANKL for 5 d. PFK15 was added on the indicated time‐points. The culture medium was changed daily during the differentiation process. Next, the cells were fixed and stained for TRAP assay. TRAP‐positive multinucleated osteoclasts were quantified. *P < .05, **P < .01 vs control. Data are presented as means ± SD of three independent experiments. Original scale bars, 500 μm

To investigate the role of PFKFB3 during osteoclastogenesis, the protein levels of PFKFB3 were measured. Western blot analysis revealed the expression of PFKFB3 increased during osteoclast differentiation (Figure 1A). To further clarify the function of PFKFB3 in osteoclastogenesis, the adenovirus encoding PFKFB3 shRNA or the control adenovirus was transfected into BMMs. The mRNA and protein expression of PFKFB3 was dramatically down‐regulated by the adenovirus encoding PFKFB3 shRNA (Figure 1B). TRAP staining results indicated genetic knockdown of PFKFB3 decreased the number and the size of mature osteoclasts (Figure 1C). Next, PFK15, a selective inhibitor of PFKFB3, was used to assess the effects of PFKFB3 inhibition on osteoclastogenesis. The CCK8 results showed that BMMs did not display a significant decline in cell viability with PFK15 treatment (Figure 1D). In addition, the differentiation of osteoclasts was significantly suppressed by PFK15 in a concentration‐dependent manner at 2‐8 μmol/L (Figure 1E). To identify which stage of osteoclastogenesis was mostly affected by PFK15, BMMs were treated with 8 μmol/L PFK15 at different time‐points during osteoclastogenesis. Our results indicated the effects of PFK15 were mainly exerted at the early stage of osteoclast differentiation (Figure 1F).

### 3.2 Blockage of PFKFB3 inhibits actin ring formation and osteoclastic bone resorption

**Figure 2:**
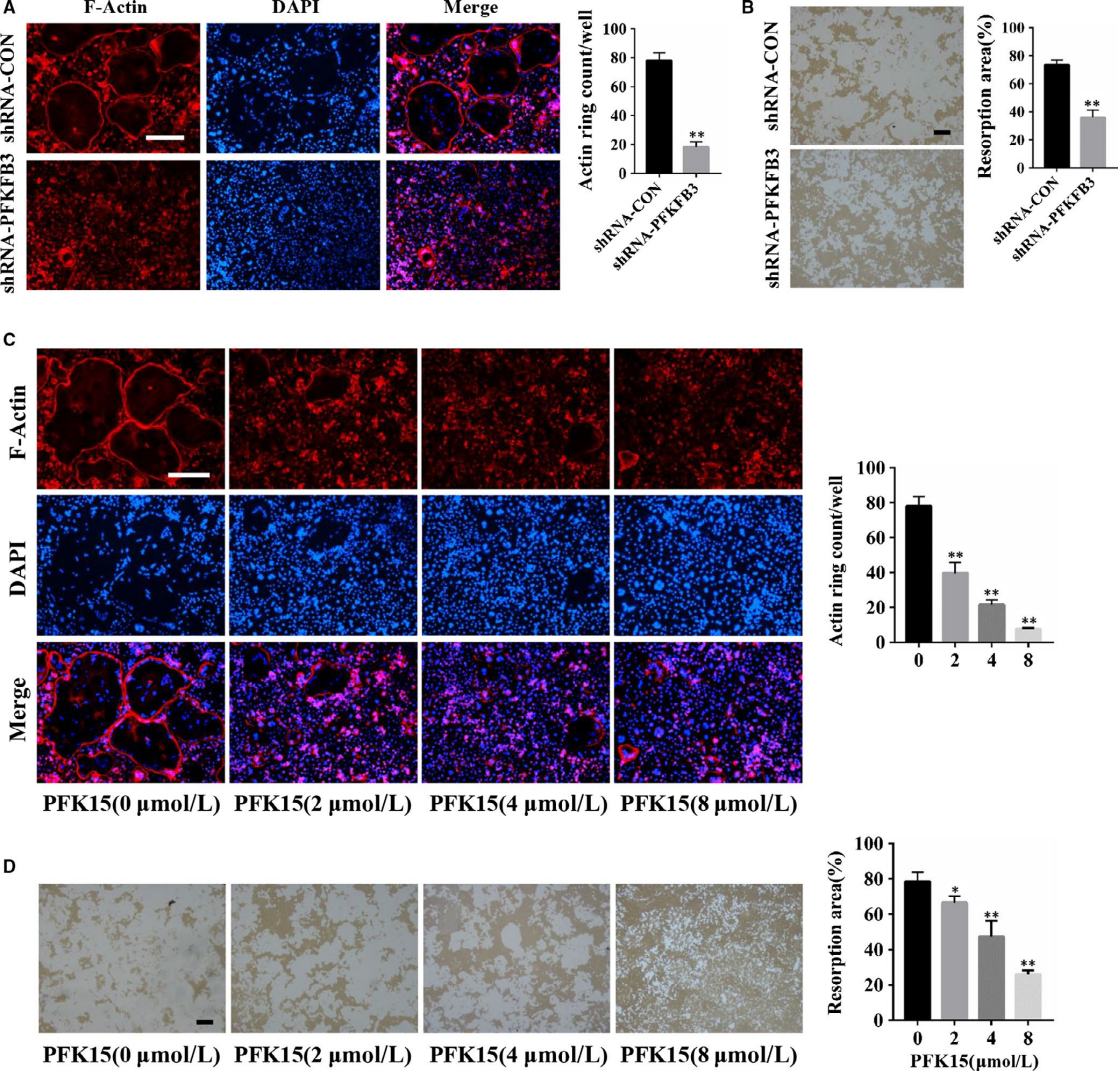
Genetic and pharmacological blockage of PFKFB3 inhibits the bone resorption activity of osteoclasts. A, B, Mature osteoclasts from BMMs were seeded in Corning osteo assay strip wells and infected with the adenovirus carrying PFKFB3 shRNA or the control adenovirus, then cultured for 2 d with M‐CSF and RANKL supplementation. F‐actin staining (A) or pit formation (B) assays were performed. C, D, Mature osteoclasts from BMMs were seeded in Corning osteo assay strip wells and treated with different concentrations of PFK15 for 2 d in the presence of M‐CSF and RANKL. F‐actin staining (C) or pit formation (D) assays were conducted. *P < .05, **P < .01 vs control. Data are presented as means ± SD of three independent experiments. Original scale bars, 250 μm

To explore the effects of PFKFB3 on osteoclast function, F‐actin ring formation assay was performed. F‐actin, a special and characteristic structure of osteoclasts, is necessary for the attachment of osteoclasts to the bone surface and subsequently bone resorption. Immunofluorescence staining showed the size and morphology of F‐actin rings was significantly impaired by the adenovirus encoding PFKFB3 shRNA (Figure 2A). Consistent with the adenovirus results, PFK15 also noticeably suppressed actin ring formation (Figure 2C). Additionally, pit formation assay was conducted to further confirm the influence of PFKFB3 on osteoclast function. The results showed resorption pits were strongly inhibited by the adenovirus carrying PFKFB3 shRNA as well as the PFKFB3 inhibitor PFK15 (Figure 2B,D).

### 3.3 PFK15 prevents OVX‐induced bone loss

**Figure 3:**
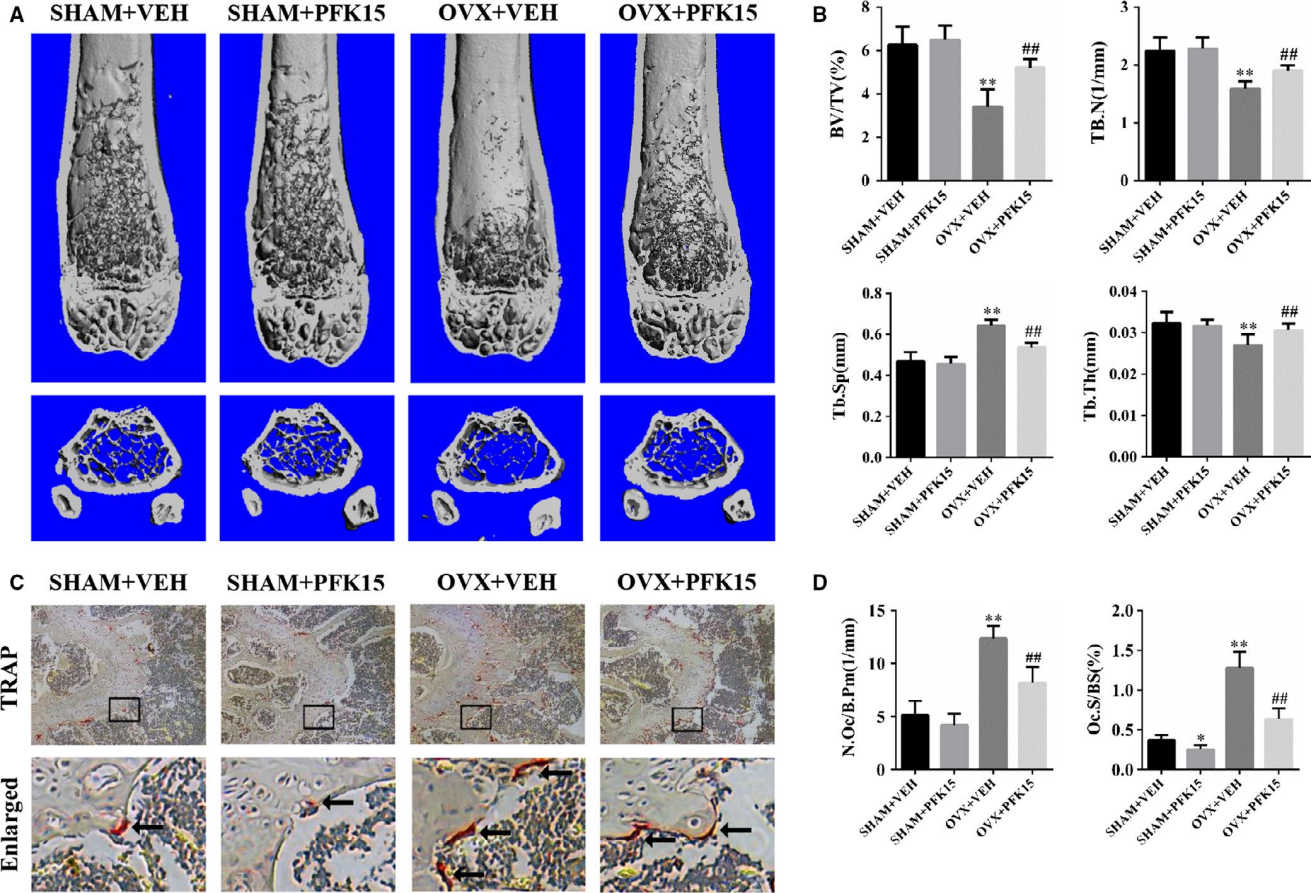
PFK15 prevents ovariectomy‐induced bone loss. A, Representative three‐dimensional μ‐CT images of distal femurs of the mice from SHAM + VEH, SHAM + PFK15, OVX + VEH, OVX + PFK15 groups. B, Bone structural parameters of the distal femurs: bone volume/tissue volume (BV/TV), trabecular numbers (Tb.N), trabecular thickness (Tb.Th) and trabecular space (Tb.Sp). C, TRAP staining of representative sections of the distal femurs in each group. D, Bone histomorphometric analyses for number of osteoclasts per bone perimeter (N.Oc/B.Pm) and osteoclast surface per bone surface (Oc.S/BS). *P < .05, **P < .01 vs the SHAM + VEH group. ^##^ *P* < .01 vs the OVX + VEH group. Data are presented as means ± SD

To mimic post‐menopausal osteoporosis, an ovariectomized murine model was used. μ‐CT scanning was conducted on the trabecular bone of the distal femurs. μ‐CT scanning results showed that the OVX mice suffered from an extensive loss of trabecular bone in comparison with the sham‐operated mice. However, PFK15 administration in the OVX mice markedly blocked the trabecular bone loss (Figure 3A). In addition, the mice from the OVX + PFK15 group displayed an increase in BV/TV, Tb.N and Tb.Th, and a decrease in Tb.Sp, compared with the mice from the OVX + VEH group (Figure 3B). To further confirm whether PFK15 prevented OVX‐induced bone loss by inhibiting osteoclast formation, TRAP staining was conducted. Our results revealed that the mice from the OVX + PFK15 group exhibited reduced numbers of red‐coloured TRAP‐positive cells at the surface of trabecular bone compared to the mice from the OVX + VEH group (Figure 3C,D).

### 3.4 PFKFB3 inhibition down‐regulates osteoclast marker gene expression

**Figure 4:**
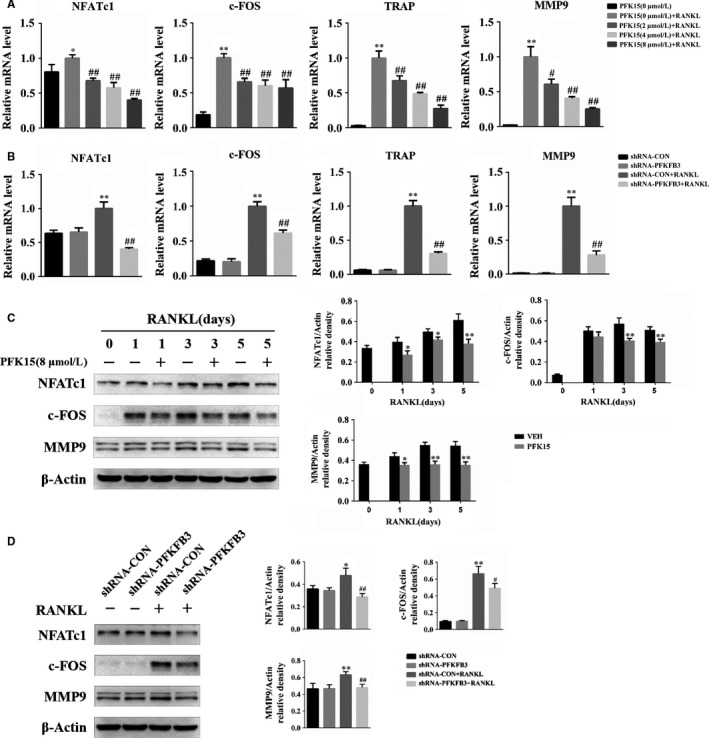
Suppression of PFKFB3 down‐regulates the expression of osteoclast marker genes. A, BMMs were treated with PFK15 of indicated concentrations in the presence of M‐CSF and RANKL for 2 d. mRNA levels of osteoclast marker genes were determined using qPCR. *P < .05, **P < .01 vs control. ^#^ *P* < .05, ^##^ *P* < .01 vs the PFK15 (0 μmol/L) + RANKL group. B, BMMs were infected with the adenovirus carrying PFKFB3 shRNA or the control adenovirus and then cultured in the presence of M‐CSF and RANKL for 2 d. mRNA expression was quantified. **P < .01 vs the shRNA‐CON group. ^##^ *P* < .01 vs the shRNA‐CON + RANKL group. C, BMMs were cultured with or without PFK15 in the presence of M‐CSF and RANKL for the indicated times. Protein levels of osteoclast marker genes were measured using Western blotting. *P < .05, **P < .01 vs control. D, BMMs were treated as described in (B), and protein expression of osteoclast marker genes was detected by Western blotting. *P < .05, **P < .01 vs the shRNA‐CON group. ^#^ *P* < .05, ^##^ *P* < .01 vs the shRNA‐CON + RANKL group. Data are presented as means ± SD of three independent experiments

Next, the expression of osteoclast‐related transcription factors and specific genes was measured. NFATc1 and c‐FOS have been reported as key transcription factors of osteoclast differentiation. TRAP and MMP‐9 are known as osteoclast marker genes. qPCR results showed the mRNA levels of NFATc1, c‐FOS, TRAP and MMP9 were enhanced by the stimulation of RANKL, but they were all dramatically reduced by PFK15 as well as the adenovirus carrying PFKFB3 shRNA (Figure 4A,B). Additionally, the expression changes of osteoclastogenic genes were further confirmed by Western blotting; our results indicated the protein expression of NFATc1, c‐FOS and MMP9, which was up‐regulated upon RANKL stimulation, was suppressed by both administration of PFK15 and transfection of adenovirus carrying PFKFB3 shRNA (Figure 4C,D).

### 3.5 PFK15 inhibits RANKL‐induced NF‐κB and MAPK activation

**Figure 5:**
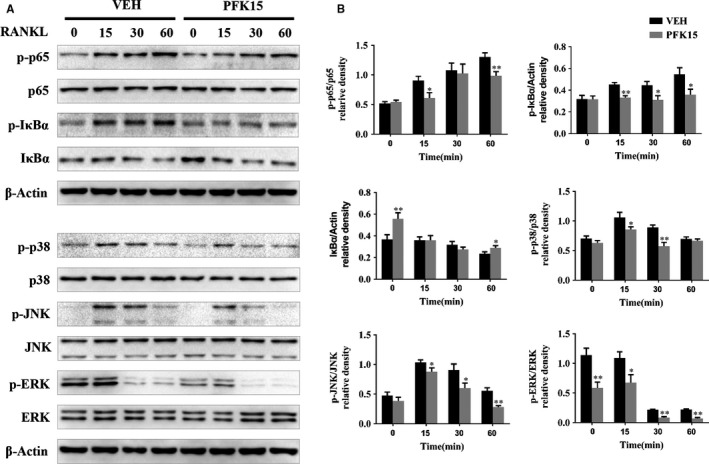
PFK15 inhibits RANKL‐induced NF‐κB and MAPK activation in BMMs. A, B, BMMs were cultured with α‐MEM in the absence of FBS for 16 h and then pre‐treated with or without PFK15 (8 μmol/L) for 24 h. Finally, BMMs were stimulated with RANKL (75 ng/mL) for the indicated times. Total and phosphorylated protein levels of NF‐κB and MAPK signalling components were analysed using Western blotting. *P < .05, **P < .01 vs control. Data are presented as means ± SD of three independent experiments

In previous studies, NF‐κB and MAPK pathways have been shown to play vital roles during osteoclast differentiation. To explore whether PFK15 inhibited osteoclastogenesis by suppressing NF‐κB and MAPK pathways, BMMs pre‐treated with DMSO or 8 μmol/L PFK15 were then stimulated with RANKL for 0, 15, 30 and 60 minutes. As shown by Western blotting, the phosphorylation levels of P65 and IκBα, the two major subfamilies of the NF‐κB pathway, were significantly attenuated by PFK15. In the MAPK pathway, the phosphorylation levels of P38, JNK and ERK were also dramatically inhibited by the administration of PFK15 (Figure 5A,B).

### 3.6 Glucose metabolic changes during RANKL‐induced osteoclastogenesis

**Figure 6:**
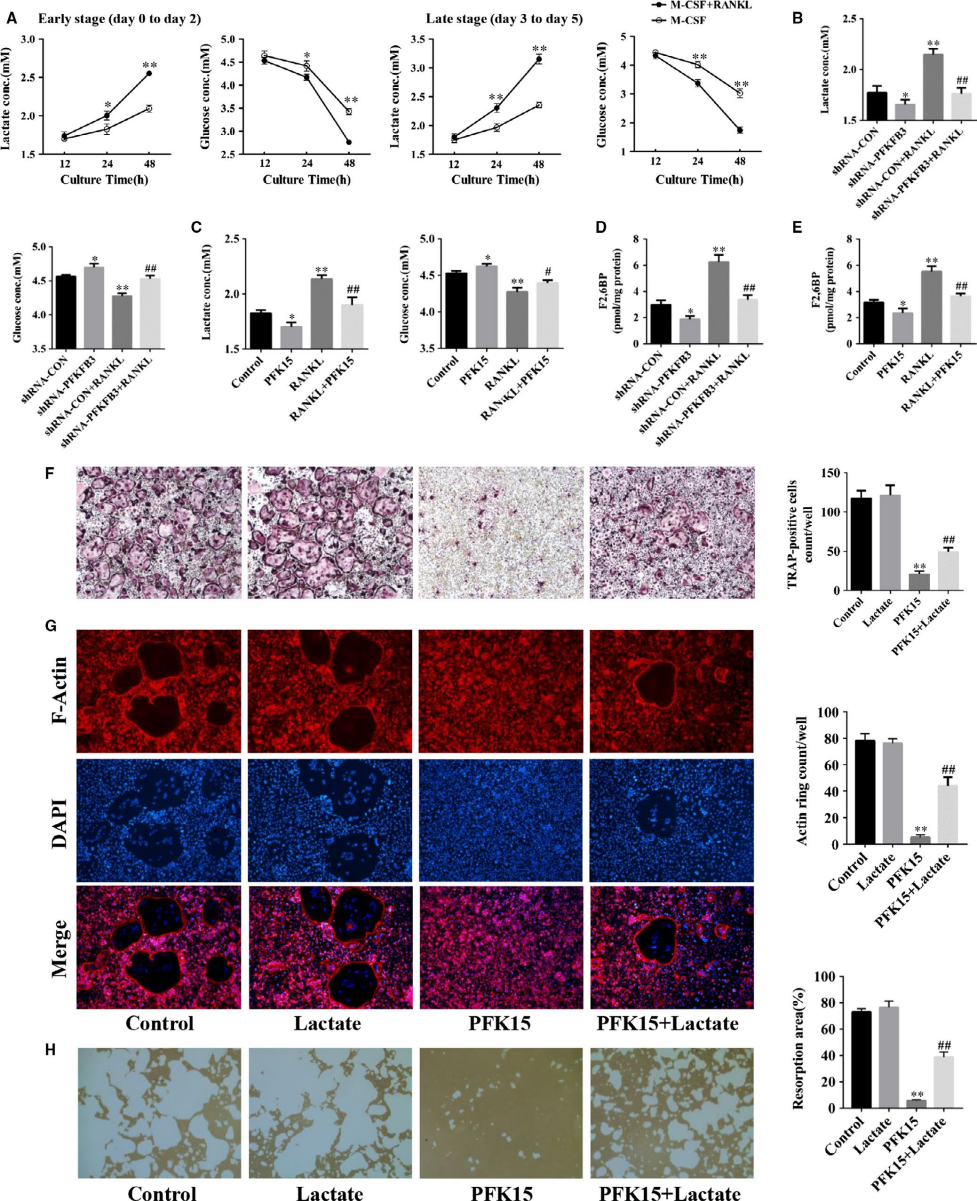
Involvement of glycolytic metabolism in PFKFB3‐mediated regulation of osteoclastogenesis. A, BMMs were stimulated with or without RANKL as indicated during early and late stages of osteoclast differentiation. Lactate and glucose concentrations in the medium were assayed using the L‐Lactate Assay kit and the Glucose Assay kit, respectively. *P < .05, **P < .01 between the two groups. B, BMMs were infected with the adenovirus carrying PFKFB3 shRNA or the control adenovirus and then cultured with or without RANKL in the presence of M‐CSF for 24 h. Lactate and glucose levels in the medium were measured. *P < .05, **P < .01 vs the shRNA‐CON group. ^##^ *P* < .01 vs the shRNA‐CON + RANKL group. C, BMMs were pre‐treated with or without PFK15 (8 μmol/L) for 24 h and then stimulated with RANKL for 24 h. Lactate and glucose levels in the media were assayed. *P < .05, **P < .01 vs control. ^#^ *P* < .05, ^##^ *P* < .01 vs the RANKL group. D, BMMs were treated as described in (B), and intracellular F2,6BP levels were detected. *P < .05, **P < .01 vs the shRNA‐CON group. ^##^ *P* < .01 vs the shRNA‐CON + RANKL group. E, BMMs were treated as described in (C), and intracellular F2,6BP levels were assayed. *P < .05, **P < .01 vs control. ^##^ *P* < .01 vs the RANKL group. F‐H, BMMs were treated with PFK15 (8 μmol/L) and L‐lactate (5 mmol/L) as indicated in the presence of M‐CSF and RANKL. TRAP staining (F), F‐actin staining (G) or pit formation (H) assays were conducted. **P < .01 vs control. ^##^ *P* < .01 vs the PFK15 group. Data are presented as means ± SD of three independent experiments

To clarify the glucose metabolic changes during RANKL‐induced osteoclast differentiation, the cellular glycolytic activity characterized by lactate accumulation and glucose consumption in growth medium during early and late stages of osteoclastogenesis was assessed. RANKL‐stimulated BMMs showed time‐dependent increases in both glucose consumption and lactate accumulation compared with RANKL‐untreated BMMs (Figure 6A). Then, the mitochondrial respiration activity represented by oxygen consumption was measured and the oxygen consumption was also enhanced upon RANKL stimulation during osteoclastogenesis (Figure S1A). Therefore, stimulation by RANKL during osteoclastogenesis induced metabolic changes to both enhanced glycolysis and accelerated mitochondrial respiration. And to further compare the role of glycolysis and mitochondrial respiration during osteoclast differentiation, the effects of rotenone, an inhibitor of mitochondrial complexes, on osteoclastogenesis were evaluated and treatment with rotenone also showed remarkable inhibitory effects on osteoclast differentiation compared with PFK15 (Figure S1C,D).

### 3.7 Glycolysis is involved in PFKFB3‐mediated regulation of osteoclastogenesis

To investigate whether glycolysis contributed to PFKFB3‐mediated regulation of osteoclastogenesis, the effects of the adenovirus carrying PFKFB3 shRNA or PFK15 on lactate accumulation and glucose consumption in growth medium were assessed, respectively. The results showed the glycolytic activity in BMMs was markedly inhibited by both transfection of adenovirus carrying PFKFB3 shRNA and administration of PFK15 (Figure 6B,C). Additionally, blockage of glycolysis by PFK15 showed a negligible inhibitory effect on oxygen consumption compared with rotenone (Figure S1B). In previous studies, PFKFB3 was shown to play a vital role in glycolysis by regulating the intercellular level of F2,6BP. Therefore, the concentration of F2,6BP in BMMs was measured in the present study. As shown in Figure 6D,E, the intracellular concentration of F2,6BP was significantly reduced by the inhibition of PFKFB3. L‐lactate is produced from pyruvate which is the end product of glycolysis, and it partially reversed the repression of osteoclastogenesis caused by PFK15 (Figure 6F). Additionally, L‐lactate also rescued the inhibitory effects of PFK15 on actin ring formation and osteoclastic resorption (Figure 6G,H). We thus inferred that glycolysis is involved in PFKFB3‐mediated regulation of osteoclastogenesis.

### 3.8 L‐lactate partially ameliorates PFK15‐mediated inhibition of NF‐κB and MAPK signalling cascades

**Figure 7:**
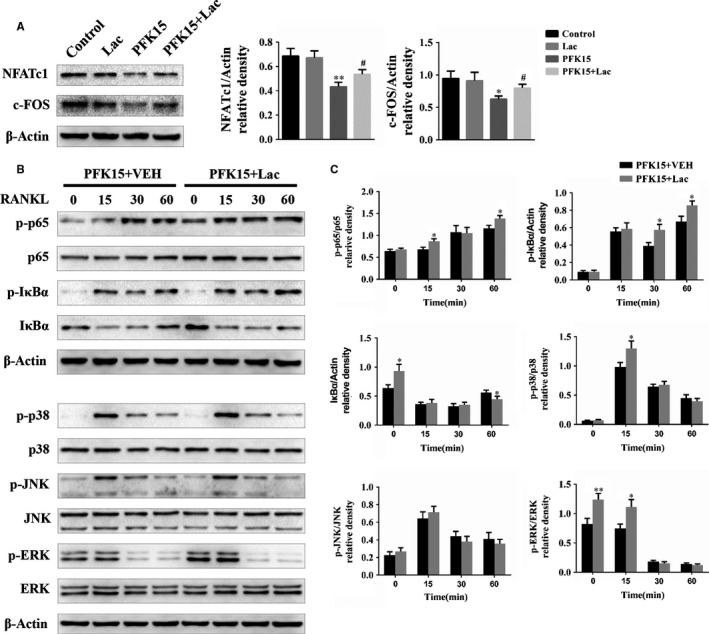
L‐lactate partially reverses PFK15‐mediated inhibition of NF‐κB and MAPK signalling cascades. A, BMMs were treated with PFK15 (8 μmol/L) and L‐lactate (5 mmol/L) as indicated in the presence of M‐CSF and RANKL for 3 d. Protein levels of NFATc1 and c‐FOS were determined by Western blotting. *P < .05, **P < .01 vs control. ^#^ *P* < .05 vs the PFK15 group. B, C, BMMs were cultured with α‐MEM in the absence of FBS for 16 h and then pre‐treated with PFK15 (8 μmol/L) and L‐lactate (5 mmol/L) for 24 h. Finally, BMMs were stimulated with RANKL (75 ng/mL) for the indicated times. Total and phosphorylated protein levels of NF‐κB and MAPK were analysed using Western blotting. *P < .05, **P < .01 vs control. Data are presented as means ± SD of three independent experiments

To further confirm the role of glycolytic metabolism in PFKFB3‐mediated regulation of osteoclastogenesis, the protein levels of crucial transcription factors during osteoclast differentiation were assessed. Western blot analysis revealed the protein expression of NFATc1 and c‐FOS, which was markedly inhibited by PFK15, was partially reversed by L‐lactate (Figure 7A). Next, the effects of L‐lactate on NF‐κB and MAPK pathways were evaluated. The decreased phosphorylation levels of P65 and IκBα in the NF‐κB signalling pathway, and P38 and ERK in the MAPK signalling pathway induced by PFK15, were partially rescued by the administration of L‐lactate (Figure 7B,C). We thus concluded that the PFK15‐mediated inhibition of NF‐κB and MAPK pathways was partially abrogated by L‐lactate.

## 4 DISCUSSION

In the current study, PFKFB3 expression was up‐regulated during RANKL‐induced osteoclast differentiation, suggesting that PFKFB3 possibly participated in the regulation of osteoclastogenesis. Next, pharmacological and genetic disruption of PFKFB3 was shown to dramatically inhibit the differentiation and function of osteoclasts. In addition, the PFKFB3 inhibitor PFK15 exhibited no apparent cytotoxicity in BMMs. Moreover, PFK15 prevented OVX‐induced bone loss in an ovariectomized murine model and abnormal health conditions were not observed in any animal. Previous studies have also shown that intraperitoneal administration of PFK15 displayed no end organ toxicity.^21^ These results suggested that targeting PFKFB3 might be a safe and promising strategy to treat oestrogen deficiency‐induced bone loss.

Our further investigation showed that blockage of PFKFB3 suppressed osteoclast specific genes such as NFATc1, c‐FOS, TRAP and MMP9. Moreover, the activation of NF‐κB and MAPK signalling cascades was also suppressed by PFK15. These results are consistent with a previous study that showed PFK15 inhibited TNFα‐induced MAPK and NF‐κB activation.^22^ NF‐κB and MAPK pathways are essential for osteoclastogenesis and are closely associated with inflammation.^23^, ^24^ A recent study demonstrated that the switch to enhanced glycolysis mediated by PFKFB3 was accompanied by the release of inflammatory cytokines in peripheral blood mononuclear cells.^25^ Another research also reported that glycolytic inhibitors of PFKFB3 reduced the expression of inflammatory cytokines in synovial fibroblasts,^26^ supporting our results that inhibition of PFKFB3 suppressed osteoclastogenesis through inflammation‐related NF‐κB and MAPK signalling pathways.

The metabolic change from oxidative phosphorylation to glycolysis in cancer cells has been reported in several studies,^27^, ^28^ and this metabolic change offered novel strategies for cancer treatment. Notably, we and others found that stimulation by RANKL during osteoclastogenesis induced a metabolic shift towards elevated glycolytic metabolism.^10^, ^11^ In addition, the mRNA levels of glycolytic marker genes, such as pyruvate kinase, phosphofructokinase and hexokinase, were reportedly up‐regulated during osteoclast differentiation.^8^ To further enrich the understanding of glucose metabolic changes during RANKL‐induced osteoclastogenesis, the role of mitochondrial respiration was investigated. We found that the mitochondrial respiration activity characterized by oxygen consumption was also enhanced upon RANKL stimulation during osteoclast differentiation and rotenone, an inhibitor of mitochondrial complexes, also dramatically suppressed RANKL‐induced osteoclastogenesis. Collectively, these results indicate that osteoclastogenesis is an active metabolic process with both increased glycolysis and mitochondrial respiration, and targeting these metabolic changes such as accelerated glycolysis or enhanced mitochondrial respiration might be promising treatments for osteoclast‐related diseases.

PFKFB3 is a key regulator in glycolytic metabolism, and its role in cancer cells has been well‐established.^29^ In recent studies, blockage of PFKFB3 suppressed the growth of various cancer cells, such as head and neck squamous cell carcinoma cells 17 and breast cancer cells.^30^ However, studies investigating the function of PFKFB3 in macrophage cells mostly focused on the relationship between glycolytic metabolism and immune defence,^31^, ^32^ and the role of PFKFB3 during osteoclast differentiation remains unclear. In our present study, pharmacological and genetic disruption of PFKFB3 suppressed glycolytic metabolism during osteoclast differentiation, which is partially in line with results from a previous study showing the PFKFB3 inhibitor attenuated lipopolysaccharide‐induced increased glycolysis in BMMs.^33^ In addition, PFK15 only showed a negligible inhibitory effect on oxygen consumption of BMMs compared with rotenone. These results revealed that PFKFB3 mainly affected glycolysis rather than mitochondrial respiration during osteoclast differentiation, and the slight inhibitory effect on oxygen consumption might also be attributed to the suppression of glycolysis. Moreover, Tang et al^34^ demonstrated that mandibular osteotomy‐induced hypoxia enhanced osteoclast differentiation and function by increasing glycolysis, supporting our result that glycolytic metabolism is essential for osteoclastogenesis.

Glycolytic metabolism regulates various physiological processes by the formation of glycolytic metabolites. L‐lactate is an essential metabolite of glycolysis, and the importance of lactate in osteoclast and bone metabolism has been reported. Lemma et al^35^ showed that lactate released from breast cancer cells promoted osteoclast activation and the formation of osteolytic lesions as a source of energy. In our present study, L‐lactate concentration in the medium was reduced by the blockage of PFKFB3 during osteoclast differentiation and administration of L‐lactate partially reversed the repression of osteoclastogenesis caused by PFKFB3 inhibition. Additionally, L‐lactate has been shown to participate in the regulation of various signalling cascades. For example, a previous study showed that lactate promoted endothelial tube formation by activating the NF‐κB signalling pathway.^36^ Furthermore, lactate contributed to PFKFB3‐driven endothelial angiogenesis by inducing AKT phosphorylation.^37^ Intriguingly, we found that administration of L‐lactate partially abrogated the inhibitory effects of PFK15 on the activation of MAPK and NF‐κB pathways. Taken together, L‐lactate served as a crucial glycolytic metabolite in PFKFB3‐mediated regulation of osteoclastogenesis.

Despite these promising results, our study has certain limitations. Balance of bone turnover is maintained by osteoclast‐mediated bone resorption and osteoblast‐mediated bone formation. Our results showed that inhibition of PFKFB3 suppressed osteoclastogenesis, but the function of PFKFB3 during osteoblast differentiation remains unknown and needs further investigation.

In summary, our results demonstrated that ablation of PFKFB3 suppressed osteoclast differentiation in vitro and prevented ovariectomy‐induced bone loss in vivo. Overall, the results indicate that blockage of glycolysis by targeting PFKFB3 represents a potential therapeutic strategy for osteoclast‐related disorders.

## CONFLICT OF INTEREST

The authors confirm that there are no conflicts of interest.

## AUTHOR CONTRIBUTIONS

J. Wang, H. Guan, Z. Fang and F. Li designed the study; J. Wang, Hui Liu, H. Kang, Q. Guo and Y. Dong conducted the study; J. Wang, Z. Lei, Huiyong Liu and Y. Sun analysed the data; J. Wang, Z. Fang and F. Li wrote the manuscript.

## Data Availability

The data that support the findings of this study are available from the corresponding author upon reasonable request.
