# Supplementary figures and images for: Inhibition of PFKFB3 suppresses osteoclastogenesis and prevents ovariectomy‐induced bone loss
Source: J Cell Mol Med. 2019 Dec 27;24(3):2294–307. doi: 10.1111/jcmm.14912 (PMC7011148; doi:10.1111/jcmm.14912)

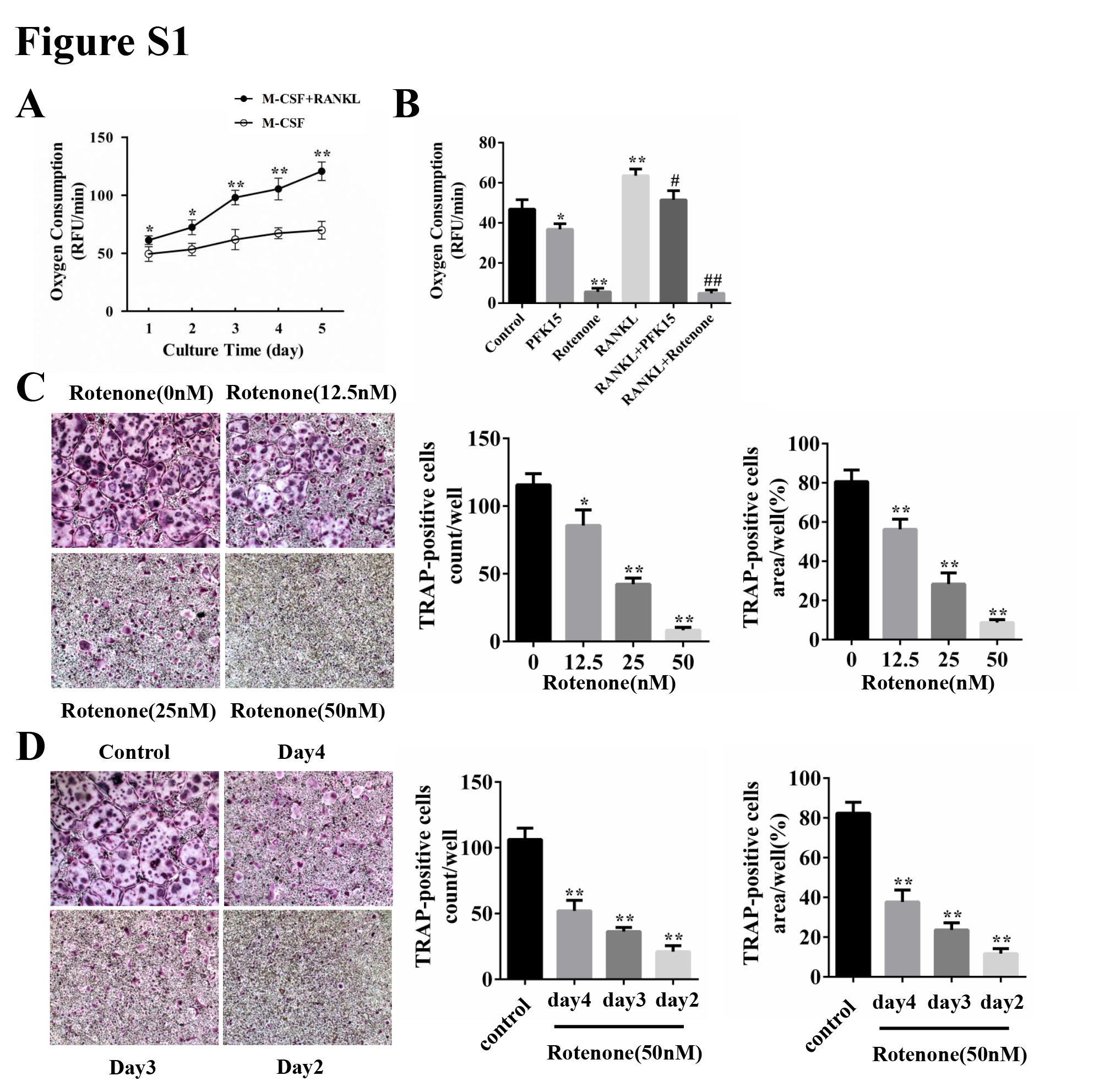

Supplement: Supplementary file 1 [file JCMM-24-2294-s001.tif]
