# Supplementary material for: Inhibition of PFKFB3 suppresses osteoclastogenesis and prevents ovariectomy‐induced bone loss
Source: J Cell Mol Med. 2019 Dec 27;24(3):2294–307. doi: 10.1111/jcmm.14912 (PMC7011148; doi:10.1111/jcmm.14912)
Supplement: Supplementary file 2 [file JCMM-24-2294-s002.docx]

**FIGURE LEGENDS**

**Figure S1.** Involvement of mitochondrial respiration during osteoclast differentiation. (A) BMMs were stimulated with or without RANKL in the presence of M-CSF for the indicated days. Oxygen consumption was detected using the extracellular oxygen consumption assay kit. **P* < 0.05, ***P* < 0.01 between the two groups. (B) BMMs were treated with RANKL, PFK15 (8 μM), and Rotenone (50 nM) as indicated for 24h. Oxygen consumption was measured using the extracellular oxygen consumption assay kit. **P* < 0.05, ***P* < 0.01 *versus* control. ^#^*P* < 0.05, ^##^*P* < 0.01 *versus* the RANKL group. (C) BMMs were cultured with various concentrations of Rotenone in the presence of M-CSF and RANKL for 5 days. TRAP-positive multinucleated osteoclasts were counted. **P* < 0.05, ***P* < 0.01 *versus* control. (D) BMMs were cultured with M-CSF and RANKL for 5 days. Rotenone was added on the indicated time points and the culture medium was changed daily during the differentiation process. TRAP-positive multinucleated osteoclasts were quantified. ***P* < 0.01 *versus* control. Data are presented as means ± SD of three independent experiments.
